# Prevalence and associated factors of corneal arcus in the geriatric population; Tehran geriatric eye study

**DOI:** 10.1186/s12886-022-02578-6

**Published:** 2022-08-31

**Authors:** Hassan Hashemi, Pooneh Malekifar, Mohamadreza Aghamirsalim, Abbasali Yekta, Hassan Mahboubipour, Mehdi Khabazkhoob

**Affiliations:** 1 Noor Research Center for Ophthalmic Epidemiology, Noor Eye Hospital, Tehran, Iran; 2 Department of Epidemiology and Biostatistics, School of Public Health, Tehran University of Medical Sciences, Tehran, Iran; 3 Eye Research Center, Tehran University of Medical Sciences, Tehran, Iran; 4 Refractive Errors Research Center, Mashhad University of Medical Sciences, Mashhad, Iran; 5 Noor Ophthalmology Research Center, Noor Eye Hospital, Tehran, Iran; 6 Department of Basic Sciences, School of Nursing and Midwifery, Shahid Beheshti University of Medical Sciences, Tehran, Iran

**Keywords:** Corneal arcus, Geriatric population, Cardiovascular risk factors, Prevalence

## Abstract

**Purpose:**

To determine the age and sex-standardized prevalence of corneal arcus and its associated factors in a geriatric population.

**Methods:**

This population-based cross-sectional study was conducted in 2019 in Tehran; the capital of Iran, using a multi-stage stratified random cluster sampling method. All participants underwent a detailed interview, blood pressure measurement, laboratory blood tests, and a complete ocular examination.

**Results:**

Three thousand three hundred ten of 3791 invitees participated in the study (response rate: 87.31%). The mean age of the participants was 69.35 ± 7.62 years (60–97 years) and 1912 (57.76%) were female. Overall, the age and sex-standardized prevalence (95% CI) of corneal arcus was 44.28% (41.21–47.39). Based on the multiple logistic regression, the odds of corneal arcus were higher in men than in women (OR: 1.51; 95% CI: 1.14–2.00); in the age group ≥ 80 years compared to the age group 60–64 years (OR: 2.44; 95% CI: 1.68–3.53), and in retired people compared to employed individuals (OR: 2.05; 95% CI: 1.31–3.21).

**Conclusion:**

The present study showed a high prevalence of corneal arcus in the geriatric population. Although various studies have reported a significant relationship between corneal arcus with blood lipid and glucose levels as well as blood pressure, these relationships were not found in the present study.

## Introduction

Corneal arcus or arcus senilis is characterized by lipid deposits appearing as a gray or white opaque ring in the peripheral cornea [1, 2]. The most accepted theory regarding corneal arcus formation is the accumulation of lipids such as cholesterol, phospholipids, triglycerides, and lipoproteins in the regions with higher corneal perfusion that have capillaries with higher permeability [3]. The presence of a corneal arcus is usually clinically insignificant; however, a corneal arcus can be an indicator of conditions such as hypercholesterolemia, hyperlipoproteinemia, or hyperlipidemia [1]. Unilateral arcus could be a sign of decreased blood flow to the unaffected eye, possibly due to carotid artery disease or ocular hypotony [4]. According to a study conducted in Singapore, the presence of corneal arcus increases the risk of cardiovascular disease even in individuals with low-risk Framingham scores [5].

Various studies have investigated the association between corneal arcus and the risk of cardiovascular disease, especially in younger age groups [6, 7]. Therefore, the presence of corneal arcus can help early diagnosis of cardiovascular disease. Older people are at risk of cardiovascular disease [8]. The prevalence of corneal arcus increases with age and reaches its highest level in older people [2, 9].

Previous studies reported the prevalence of corneal arcus among different populations in a wide range from 5% in Taiwan to 73.2% in Singapore [2, 10]. However, most previous studies were hospital-based with a wide age range. The prevalence of corneal arcus varies by ethnicity. A higher prevalence of corneal arcus has been reported in people of African and Southeast-Asian descent compared to individuals of white descent [3]. The Shahroud Eye Cohort Study conducted in Iran, reported a 23.3% prevalence of corneal arcus in the age group 40–65 years [11]. To the best of our knowledge, no population-based study has specifically examined the prevalence of corneal arcus in the geriatric population.

Moreover, the associated factors of corneal arcus have not yet been fully identified. Apart from age and race, sex was also reported as an associated factor of corneal arcus in some studies so that the prevalence of arcus in men was significantly higher than in women [2, 12]. However, the association of various other variables with corneal arcus has not been investigated. Identifying these related factors can help to understand the nature of the corneal arcus better. According to the above, the present study aimed to determine the prevalence of corneal arcus and its related factors in a population of Iranian old adults.

## Methods

### Design and sampling

This report is a part of the Tehran Geriatric Eye Study (TGES); a population-based cross-sectional study that conducted in 2019 in Tehran, the capital of Iran, using a multi-stage stratified random cluster sampling. The target population of TGES was all residents 60 years of age and above in Tehran city. Since the main objective of TGES was to investigate visual impairment, the sample size was calculated based on the prevalence of visual impairment in Tehran. The sample size for a rate of 5.2%, a precision level of 0.01, a 95% confidence level, a 1.5 design effect, and a non-response rate of 10% was calculated as 3200.

In the first step, each of the 22 districts of Tehran was considered a stratum. The over 60-year population of Tehran was obtained from the Statistical Center of Iran. Then, the block-by-block map of each district was provided and each block was considered a cluster. Next, 160 blocks each containing 20 individuals were randomly selected from all 22 districts and the number of clusters in each district was proportional to its population.

All individuals 60 years of age and above were invited to participate in the study after explaining the goals and steps of the study. This process continued until the completion of the required sample size in each cluster. If the eligible individuals in the last household of a cluster were more than one person, this cluster could include more than 20 individuals. The study participants were transferred to the study site free of charge on a pre-scheduled day. The study site was a specialty eye hospital in Tehran, Iran (Noor Eye Hospital). After the participants arrived at the study site, a face-to-face interview was performed to collect complete demographic, socio-economic, and anthropometric information, as well as the history of previous ocular examinations, ocular and systemic diseases, previous ocular surgery, and ocular trauma. The next steps included measurement of height and weight, blood pressure, blood tests, and a complete ocular examination.

### Examinations and definitions

To determine blood pressure, two measurements were performed at intervals of 3 min. If there was a difference of more than 5 mm Hg in diastolic pressure or 10 mm Hg in systolic blood pressure, the third measurement was performed and the average of these measurements was considered as the final result. If the difference was less than 5 mmHg, the average of the first two measurements was considered as blood pressure.

Hypertension (HT) was defined based on a previous diagnosis or a systolic pressure ≥ 140 mm Hg or a diastolic pressure ≥ 90 mm Hg [13]. Dyslipidemia was defined as taking lipid control drugs or low-density lipoprotein (LDL) > 160 mg/dl or high-density lipoprotein (HDL) < 40 mg/dl or cholesterol > 240 mg/dl or triglycerides > 150 mg/dl in a blood test [14]. Hemoglobin A1c (HbA1c) ≥ 6.5% or a history of glycemic control drugs was defined as hyperglycemia [15]. Ocular examinations included measurement of uncorrected and best-corrected distance visual acuity using a LED visual acuity chart (Smart LC 13, Medizs Inc., Korea) at 6 m, objective refraction by an auto-refractometer (ARK-510A, Nidek Co. 42 LTD, Aichi, Japan), subjective refraction, and ocular health examination of the anterior and posterior ocular segments using a slit-lamp biomicroscope (Haag-Streit AG, Bern, Switzerland) and + 90 dipoter (D) lens. The diagnosis of corneal arcus was performed based on the slit-lamp examination by an ophthalmologist. The corneal arcus was defined as the presence of hazy white-colored lipid in the corneal stroma, mostly in the superior or inferior pole, separated from the limbus by a transparent rim, sometimes forming a complete (360 degrees) ring [16].

### Statistical analysis

To calculate the age and sex-standardized prevalence of corneal arcus, the population of Tehran in 2016 (the last national census) was prepared by age and sex. The sample was then standardized accordingly, and the prevalence of corneal arcus was estimated with a 95% confidence interval (CI). The cluster effect was considered to correct the standard error. To investigate the relationship between various study variables and corneal arcus, a simple logistic regression was used. All variables with a *P*-value < 0.05 in the simple logistic regression were entered into a multiple logistic regression model.

We first analyzed the association between the independent variables to check multicollinearity. If there was a strong association between the independent variables, only one of them was included in the multiple model. All analyzes were performed with Stata-11 software. A *P* value < 0.05 was considered statistically significant.

### Ethical issues

Informed consent was obtained from all participants. The principles of the Helsinki Declaration were followed in all stages of the study. The protocol of the study was approved by the Ethics Committee of the National Institute for Medical Research Development (NIMAD) under the auspices of the Iranian Ministry of Health (ethics code: IR.NIMAD.REC.1397.292).

## Results

3310 of 3791 invitees participated in this study (response rate: 87.31%). The mean age of the participants was 69.35 ± 7.62 years (60–97 years) and 1912 (57.76%) were female. Overall, the standardized prevalence (95% CI) of corneal arcus was 44.28% (41.21–47.39). Table 1 presents characteristics of the study participants.

**Table 1:** The distribution of participants’ characteristics in this study

Variables	n (%)
Total	1466 (44.28)
Involved Eye	
Unilateral	68 (4.6)
Bilateral	1398 (95.4)
Gender	
Female	1912 (57.8)
Male	1398 (42.2)
Age Group	
60—64	1165 (35.2)
65—69	954 (28.8)
70—74	634 (19.2)
75—79	313 (9.5)
80 and more	244 (7.37)
Education Level	
Illiterate	448 (13.5)
Primary School	1013 (30.6)
Guide School	616 (18.6)
High School	820 (24.8)
College	413 (12.5)
Socio economic status	
Low	1169 (35.3)
Middle	1045 (31.6)
High	1096 (33.1)
Occupation	
Employed	109 (3.3)
Retired	1442 (43.6)
Unemployed/ disabled	137 (4.1)
Housekeeper	1622 (49.0)
Alcohol	
No	3123 (94.4)
Yes	187 (5.6)
Smoking	
No	2877 (86.9)
Yes	433 (13.1)
Hyperglycemia	
No	1469 (44.4)
Yes	425 (12.8)
Dyslipidemia	
No	487 (14.7)
Yes	785 (23.7)
Hypertension (systolic pressure)	
No	1844 (55.7)
Yes	1466 (44.3)
Hypertension (diastolic pressure)	
No	2773 (83.8)
Yes	537 (16.2)

The standardized prevalence of unilateral and bilateral corneal arcus was 2.03% (95% CI: 1.49 to 2.75) and 42.25% (95% CI: 39.26–45.31), respectively. Table 2 presents the prevalence of corneal arcus by different study variables. As seen in Table 2, the prevalence of corneal arcus in men and women was 47.76% (95% CI: 44.18–51.35) and 40.84% (95 CI%: 37.15–44.65), respectively. The prevalence of corneal arcus was significantly higher in men than in women. The prevalence of corneal arcus increased with age, from 33.73% (95% CI: 30.20–37.45) in the age group 60–64 years to 55.60% (95% CI: 46.75–64.11) in the age group 80 years and above. The prevalence of corneal arcus decreased with increasing level of education from 47.77% (95% CI: 42.32–53.27) in illiterate persons to 40.49% (95% CI: 35.94–45.20%) in individuals with high school education. However, the prevalence showed an increase in individuals with university education and reached 46.73% (95% CI: 40.92–52.63). The prevalence of corneal arcus decreased with increasing socioeconomic level from 45.77 (95% CI: 41.70–49.89) in the low socioeconomic status group to 39.69 (95% CI: 35.45–44.09) in the high socioeconomic status group. The prevalence of corneal arcus was different among various occupational groups so that the highest and lowest prevalence was observed in disabled/ unemployed (48.12%, 95% CI: 39.30—57.06) and employed (28.44%, 95% CI: 20.62—37.81) participants, respectively. Moreover, the prevalence of corneal arcus was higher in smokers (47.34%, 95% CI: 42.60–52.14) than in non-smokers (42.30%, 95% CI: 39.19–45.48%). As Table 2 shows, the prevalence of corneal arcus in individuals with hyperglycemia, dyslipidemia, and hypertension was not significantly different from that of other individuals.

**Table 2:** The age and sex-standardized prevalence and results of simple logistic regression analysis for the association between corneal arcus and different study variables

Variables	Prevalence% (95% CI)	Odds ratio (95% CI)	*p*—value
Total ^a^	44.28 (41.21—47.39)	-	-
Involved Eye			
Unilateral	02.03 (01.49—02.75)	-	-
Bilateral	42.25 (39.26—45.31)	-	-
Gender			
Female	40.84 (37.15—44.65)	1	-
Male	47.76 (44.18—51.35)	1.50( 01.29—1.75)	< 0.001
Age Group			
60—64	33.73 (30.20—37.45)	1	-
65—69	42.55 (37.99—47.24)	1.48 (01.24—1.78)	< 0.001
70—74	51.69 (47.10—56.25)	2.25 (01.82—2.77)	< 0.001
75—79	55.19 (49.37—60.86)	2.57 (02.00—3.31)	< 0.001
80 and more	55.60 (46.75—64.11)	3.05 (02.22—4.19)	< 0.001
Education Level			
Illiterate	47.77 (42.32—53.27)	1	-
Primary School	42.65 (37.94—47.49)	0.81 (00.63—1.05)	0.115
Guide School	40.75 (36.29—45.36)	0.75 (00.57—0.98)	0.038
High School	40.49 (35.94—45.20)	0.74 (00.56—0.98)	0.038
College	46.73 (40.92—52.63)	0.96 (00.70—1.31)	0.792
Socio economic status			
Low	45.77 (41.70—49.89)	1	-
Middle	43.25 (39.10—47.50)	0.90 (00.75—1.08)	0.274
High	39.69 (35.45—44.09)	0.78 (00.63—0.97)	0.027
Occupation			
Employed	28.44 (20.62—37.81)	1	-
Retired	47.09 (43.43—50.78)	2.24 (01.46—3.43)	< 0.001
Unemployed/ disabled	48.12 (39.30—57.06)	2.33 (01.31—4.16)	0.004
Housekeeper	39.77 (35.99—43.67)	1.66 (01.07—2.57)	0.023
Alcohol			
No	42.65 (39.59—45.77)	1	-
Yes	48.13 (40.39—55.95)	1.25 (00.91—1.72)	0.173
Smoking			
No	42.30 (39.19—45.48)	1	-
Yes	47.34 (42.60—52.14)	1.23 (01.02—1.48)	0.033
Hyperglycemia			
No	43.28 (39.62—47.01)	1	-
Yes	43.07 (37.21—49.14)	0.99 (00.79—1.25)	0.943
Dyslipidemia			
No	42.70 (38.53—46.98)	1	-
Yes	43.90 (39.66—48.22)	1.05 (00.86—1.28)	0.623
Hypertension (systolic pressure)			
No	41.62 (38.30—45.02)	1	-
Yes	44.69 (41.14—48.30)	1.13 (00.99—1.30)	0.073
Hypertension (diastolic pressure)			
No	42.75 (39.61—45.95)	1	-
Yes	44.16 (39.21—49.23)	1.06 (00.87—1.30)	0.575

^a^ Standardized to Tehran 2016 population census; CI: Confidence Interval; SES: Socioeconomic status

Table 3 shows the results of multiple logistic regression for the relationship between corneal arcus and study variables. Among the studied variables, sex, age, education level, socioeconomic status, occupation, and smoking had a significant relationship with corneal arcus and had the criterion of entering multiple model. Based on the logistic regression model, the odds of corneal arcus in males was significantly higher than in females (OR: 1.51; *P* = 0.005). Advancing age significantly increased the risk of corneal arcus so that the odds of corneal arcus was 2.44 times in individuals 80 years and above compared to individuals aged 64–60 years (*P* < 0.001). The odds ratio of corneal arcus was also different among occupation groups so that, the odds of corneal arcus in housekeepers was 2.2 (95% CI: 1.34–3.61) times greater than employed people and retired had 2.05 (95%CI 1.31–3.21) times the odds of corneal arcus compared to employed people.

**Table 3:** The association between corneal arcus and study variables by multiple logistic regression

	First model		Last model	
Variables	Odds ratio (95% CI)	*p*—value	Odds ratio (95% CI)	*p*—value
Gender				
Female	1	-	1	
Male	1.75 (1.34—2.27)	< 0.001	1.51 (1.14–2.00)	0.005
Age Group				
60—64	1	-	1	-
65—69	1.43 (1.19—1.72)	< 0.001	1.44(1.19 -1.73)	< 0.001
70—74	2.15 (1.72—2.70)	< 0.001	2.09(1.68 -2.59)	< 0.001
75—79	2.31 (1.76—3.02)	< 0.001	2.39(1.84 -3.1)	< 0.001
80 and more	2.75 (1.97—3.83)	< 0.001	2.44(1.68 -3.53)	< 0.001
Education Level				
Illiterate	1	-	NS	
Primary School	0.88 (0.67—1.16)	0.371		
Guide School	0.84 (0.63—1.12)	0.232		
High School	0.99 (0.73—1.34)	0.950		
College	1.29 (0.91—1.83)	0.152		
SES				
Low	1	-	NS	
Middle	1.00 (0.82—1.22)	0.976		
High	0.86 (0.68—1.08)	0.191		
Occupation				
Employed	1	-	1	-
Retired	2.00 (1.31—3.07)	0.001	2.05(1.31 -3.21)	0.002
Unemployed/ disabled	1.70 (0.94—3.10)	0.081	1.59(0.87 -2.89)	0.127
Housekeeper	2.70 (1.65—4.42)	< 0.001	2.2(1.34 -3.61)	0.002
Smoking				
No	1	-	NS	
Yes	1.04 (0.84—1.28)	0.723		

*CI* Confidence Interval *NS* Not significant

Other variables including education level, socioeconomic level, and smoking status did not show a statistically significant relationship with the corneal arcus in the multiple regression (all *P* values > 0.05).

## Discussion

In the present study, we investigated the age and sex-standardized prevalence of corneal arcus in a geriatric population 60 years and above in Tehran, Iran. As mentioned earlier, the corneal arcus does not significantly affect vision but has been considered in various studies due to its association with cardiovascular diseases. The specific prevalence of this condition in the older individuals is one of the topics that has received less attention. In this study, about half of the participants (44.28%) had corneal arcus, of which bilateral type was more common. Table 4 shows the prevalence of corneal arcus in previous studies. [1, 2, 9–12, 17–19].

**Table 4:** Summary of previous studies regarding corneal arcus

First Author (Ref)	Population/Country	Age Group	Sample size	Year Of Publish	Prevalence (%)
Chen H-T [10]	Taiwanese	30–60	2363	2009	5
Vurgese S [9]	Indian rural	> 30	4711	2011	10.7
Hashemi H [11]	Iranian	40–64	6311	2014	23.3
Sakamoto A [17]	Japanese	> 50	1088	2004	30
Sakamoto A [17]	Iceland	> 55	846	2004	51.1
Rouhiainen P [19]	Eastern Finnish	-	447	1993	51.4
Wu R [2]	Singapore (Malay Eye Study)	40–80	3260	2010	73.2
Sandar M [12]	Singapore (Malay Eye Study)	40–80	3280	2007	58.1
Chua BE [1]	Blue Mountains Eye Study	> 49	3654	2004	64.8

As Table 4 shows, the prevalence of corneal arcus varies from 5% in Taiwan to 73.2% in Singapore [2, 10].

However, these differences and variations should be interpreted with caution as different causes can be involved. Factors such as demographic and racial differences, differences in blood factors including triglyceride and cholesterol levels in the studied populations [20, 21], and differences in the examination methods and diagnostic criteria [22] may contribute to the discrepancy in prevalence reported among different studies.

The difference in age distribution is one of the important reasons for the discrepancies in the reported prevalence of corneal arcus among different studies [23]. Based on the information in Table 4, it appears that the prevalence of corneal arcus is lower in studies with younger age groups. In a study conducted on individuals aged 40 to 64 years in Iran (Shahroud), the prevalence of corneal arcus was 23.3%, which is lower than the present study [11]. Given the differences in the studied age groups, this difference is expected.

In the present study, the prevalence of corneal arcus in men (47.76%) was higher than in women (40.84%). This finding is in line with other similar studies conducted in this regard [2, 12, 17, 18]. This could be due to the difference in risk factors such as blood lipid or glucose levels, and blood pressure between the two sexes. Moreover, the prevalence of corneal arcus increased with advancing age and reached 55.60% in the age group ≥ 80 years. This strong association between corneal arcus and age has been observed in several studies [23, 24]. Although the exact etiology of the corneal arcus is unclear, structural and degenerative changes in corneal stroma along with increased blood lipid levels may contribute to this finding.

In this study, the risk of corneal arcus was different among groups with different occupational status, so that the odds of corneal arcus in unemployed and disabled individuals was 1.7 times that of employed people. Employed persons also had a lower odds of corneal arcus than housekeepers and retirees. Previous studies have focused less on the relationship between employment status and the corneal arcus, and little information is available in this regard. Bulpitt et al. similarly found that people with lower occupation levels had a higher prevalence of corneal arcus than those with higher occupation levels [25]. It is difficult to explain this relationship, but it seems to be due to more physical activity in working people, which is associated with a lower risk of corneal lipid deposition.

On the other hand, since most homemakers are women, it seems that the prevalence difference between this occupational group and other occupational groups is due to age. Therefore, more research is necessary to pass judgment on the prevalence difference (29). Moreover, lifestyle differences in retired people and even their occupational history may be possible reasons for this finding.

In this study, no significant association was found between corneal arcus and socioeconomic status, level of education and other demographic factors. Although some studies have reported a significant relationship between corneal arcus with smoking and alcohol consumption, these associations were not found in the present study [2, 5, 26]. Although the relationship between corneal arcus and smoking was statistically significant in the univariate analysis, it was not significant in the multiple regression model.

Hypertension and hyperglycemia were other study variables that were not significantly associated with corneal arcus in the present study. In this study, no significant association was found between dyslipidemia and corneal arcus. Although several studies have shown that corneal arcus can be a sign of dyslipidemia as a risk factor for cardiovascular disease in individuals under 50 years of age, this association was not observed in the geriatric population [10, 21]. Corneal arcus does not seem to be an independent risk factor for cardiovascular disease in the older people, and only its presence in younger individuals could be a sign of lipid abnormalities, which needs further investigation [27]. Corneal arcus is more of a physiological finding than a pathological condition in the older people.

On the other hand, lack of statistically significant difference between corneal arcus and dyslipidemia could be due to the interaction of ethnicity and genetics with these factors in the occurrence of corneal arcus, which merits attention.

The strengths of the present study include the study design and a large sample size. However, there are some limitations. Using fasting blood sugar instead of HbA1c could provide a better estimate of hyperglycemia. Also, the use of anterior segment imaging instead of slit-lamp examination could better diagnose corneal arcus.

## Conclusion

The present study showed a high prevalence of corneal arcus in the geriatric population. Although various studies have reported a significant relationship between corneal arcus with blood lipid and glucose levels as well as blood pressure, these relationships were not found in the present study.

## Data Availability

The datasets used and/or analysed during the current study available from the corresponding author on reasonable request.
